# Incidence of extreme hyperglycemia and hypoglycemia in hospitalized patients during the month of July in teaching hospitals

**DOI:** 10.3402/jchimp.v2i2.17469

**Published:** 2012-07-16

**Authors:** Mansur Shomali, Malek Cheikh

**Affiliations:** Department of Medicine, MedStar Union Memorial Hospital, Baltimore, MD, USA

**Keywords:** hyperglycemia, hypoglycemia, hospital, residents, insulin, diabetes

## Abstract

**Introduction:**

Blood glucose control has been found to be an important component in the care of hospitalized patients. Maintaining blood glucose within a target range using insulin intensively is a challenging task for physicians and requires skill and experience. We hypothesized that there may be more hyper- and hypoglycemia in July in teaching hospitals when new resident physicians begin their training.

**Methods:**

We reviewed point-of-care blood glucose data from hospitalized patients at four community teaching hospitals for 2010. We defined severe hypoglycemia as blood glucose < 41 mg/dL and severe hyperglycemia as blood glucose > 399 mg/dL. Occurrence of hyper- and hypoglycemic events was assessed overall at the particular hospital globally and based on individual nursing units. Monthly occurrence rates were compared against the annual mean for that unit.

**Results:**

The occurrence of hyper- and hypoglycemic events in July 2010 did not differ from the mean annual percentage of events at the applicable hospital. However, when the data were analyzed by the nursing unit, these extreme glucose events were significantly more common in 4 of the 11 units studied. Three of those four units were resident teaching units.

**Conclusions:**

These data suggest that there is some potential for increased risk of extreme hyper- and hypoglycemia at teaching hospitals in July, when new residents begin training.

Blood glucose control is an important component in the care of hospitalized patients, and some studies have shown that the extremes of blood glucose confer risk for patients in hospital settings. Studies have shown that hyperglycemia increases mortality caused by myocardial infarctions (1). In a randomized controlled trial, maintaining blood sugar at or below 110 mg/dL decreased mortality by 40% in critically ill surgical patients (2). Other studies suggest that poor glucose control is an independent risk factor for nosocomial infections (3) and that hyperglycemia worsens outcomes from community acquired pneumonia (4) and stroke (5). Hypoglycemia can be associated with increased risk of mortality (6) and can increase the length of hospital stay (7). The incidence of hypoglycemia ranged between 4 and 11% in one survey of 44 US hospitals (8).

Treating hospitalized patients with diabetes can be very challenging. Oral agents in the hospital are often discontinued or are ineffective. Blood sugar control is managed by administering long- and fast-acting insulin. Because of a number of factors, such as the stress response to illness, variable nutrient intake, and variations in sensitivity to insulin, dosing insulin in these patients is often difficult and needs to be adjusted daily.

Maintaining glycemic control using insulin intensively is challenging and requires skill and experience. It is possible that glycemic control might not achieve desired levels when managed by inexperienced physicians. If this is the case, we would expect to see a decrease in patient glycemic control when new resident physicians begin training. We hypothesized that blood glucose control would not be so good in July, when most new residents begin training, compared with other months.

## Methods

We reviewed data from four community teaching hospitals, which are part of a regional health system in Baltimore, Maryland, for the calendar year 2010. The data were collected from point-of-care testing glucometers (Accu-Chek, Rouche), which are located at the nursing stations and transmit data to the medical laboratory. The data were analyzed using commercial software (Medical Automation Systems, Inc., Charlottesville, VA). We defined severe hypoglycemia as blood glucose < 41 mg/dL and severe hyperglycemia as blood glucose > 399 mg/dL.

To normalize for variation in the total number of patients each month and at different hospitals, the number of hyper- and hypoglycemia episodes was divided by the total number of blood glucoses measured in a unit or hospital in a given period.

At hospitals 2 and 3, resident physicians and other providers participated in the care of patients on all of the nursing units. At hospitals 1 and 4, patients were admitted to nursing units, which could be categorized as physicians, or non-teaching units, where orders were written predominantly by nurse practitioners and physician assistants. Data were tabulated for 11 of these units at hospitals 1 and 4 based on calendar month, and the percentages were compared with the annual mean for that unit. Units were identified during the month of July when the proportion of events exceeded two standard deviations above the annual mean for that particular unit.

## Results

**Fig. 1:**
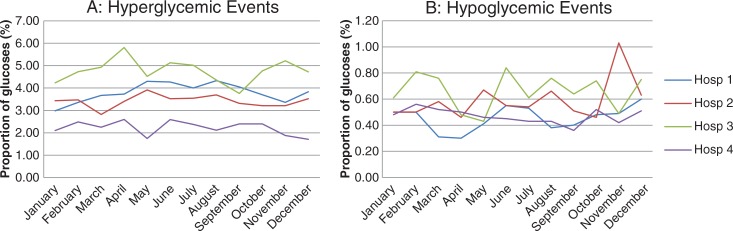
Graph shows percentage of hyperglycemic (A) and hypoglycemic (B) events at four hospitals in 2010.

**Fig. 2:**
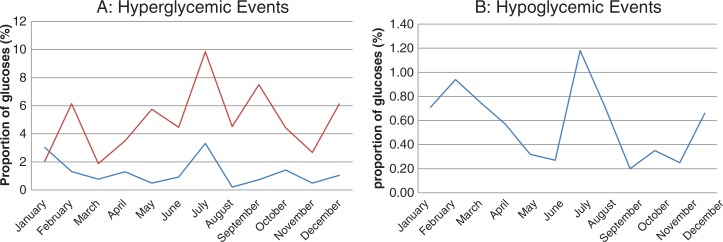
Graph shows percentage of hyperglycemic (A) and hypoglycemic (B) events in three resident training nursing units in 2010.

A total of 578,608 blood glucose measurements were taken at four hospitals. The proportion of glucose events for all four hospitals is shown in Fig. 1. Percentage of hyper- or hypoglycemic events was not elevated in July at any of the hospitals. Two individual units were found to have hyperglycemia events exceeded two standard deviations greater than the annual mean for that unit during the month of July (Table 1). Both units with elevated levels were resident teaching units. No other month in 2010 had a percentage of extreme blood glucose control events more than two standard deviations than the mean in these two units. Two units had hypoglycemia events in July more than two standard deviations above the annual mean for that unit (Table 2). One of these units was a resident teaching unit. No other month showed a high percentage of hypoglycemia events in these two units. Fig. 2 shows the percentage of hyperglycemic (A) and hypoglycemic (B) events in the three resident training nursing units in 2010 where significant differences were found in July.

**Table 1:** Proportion of hyperglycemic events in 11 nursing units at two community teaching hospitals

	Unit 1	Unit 2	Unit 3	Unit 4	Unit 5	Unit 6	Unit 7	Unit 8	Unit 9	Unit 10	Unit 11
January	3.76	1.51	2.68	0.73	0.34	0	0	3.02	0	2.05	3.01
February	2.04	0.31	8.45	0.96	0.6	1.4	0.38	1.3	0	6.14	1.66
March	1.88	3.51	2.19	1.46	0.87	1.49	0	0.76	0	1.87	1.05
April	2.95	4.31	2.81	0.82	1.11	1.05	1.18	1.29	0	3.51	7.2
May	1.92	1.71	2.76	0.15	1.67	0	0.62	0.48	0	5.74	1.41
June	1.9	3.48	2.65	2.17	0.28	0	0.35	0.91	0	4.46	4.17
July	1.94	2.44	3.15	0	0	0	0.81	3.31*	0	9.85*	4.1
August	2.42	2.85	0.31	0.53	1.43	0	0.22	0.2	0	4.52	3.41
September	2.72	3.61	2.61	2.35	0	0.79	0.32	0.73	0	7.5	3.11
October	2.49	3.02	3.29	2.6	0	0	1.36	1.41	0	4.42	2.05
November	2.27	1.83	1.91	2.93	0	0	0.15	0.48	0	2.66	1.25
December	1.47	2.44	0.63	0.15	0	0	0.26	1.03	0	6.12	5.33
Mean	2.3	2.6	2.8	1.2	0.5	0.4	0.5	1.1	0	4.5	3.1
Standard deviation	0.6	1.1	2.0	1.0	0.6	0.6	0.4	0.8	0	1.8	1.9

* Percentage is more than two standard deviations above the annual mean for that unit.

**Table 2:** Proportion of hypoglycemic events in 11 nursing units at two community teaching hospitals

	Unit 1	Unit 2	Unit 3	Unit 4	Unit 5	Unit 6	Unit 7	Unit 8	Unit 9	Unit 10	Unit 11
January	0.40	0.71	1.14	0.31	0.00	0.00	0.00	0.25	7.11	0.16	0.30
February	0.23	0.94	0.68	0.60	0.60	0.00	0.00	0.54	3.59	0.00	0.00
March	0.43	0.75	0.46	0.27	0.29	0.00	0.00	0.69	2.93	0.16	0.42
April	0.68	0.57	0.53	0.41	0.28	0.00	0.43	0.56	0.88	0.00	0.00
May	0.35	0.32	0.61	0.00	0.00	0.00	0.64	0.88	3.57	0.00	0.28
June	0.35	0.27	0.61	0.00	0.00	0.00	0.00	0.91	3.57	0.00	0.00
July	0.11	1.18*	0.00	0.00	0.88*	0.00	0.27	0.28	0.44	0.16	0.29
August	0.35	0.71	0.00	0.53	0.29	0.00	0.45	0.59	1.48	0.75	0.31
September	0.18	0.20	0.70	0.34	0.00	0.00	0.32	0.00	1.39	0.50	0.00
October	0.64	0.35	0.24	0.00	0.34	0.00	0.15	0.53	3.06	0.00	0.88
November	0.68	0.25	0.43	0.00	0.00	0.00	0.30	0.19	1.04	1.06	2.49
December	0.70	0.66	0.00	0.15	0.29	0.00	0.13	0.28	2.64	0.00	1.23
Mean	0.4	0.5	0.5	0.2	0.2	0	0.2	0.5	2.6	0.2	0.5
Standard deviation	0.2	0.3	0.3	0.2	0.2	0	0.2	0.3	1.8	0.4	0.7

* Percentage is more than two standard deviations above the annual mean for that unit.

## Discussion

Percentage of hyper- or hypoglycemic events was not higher overall in July in the four hospitals studied, but percentages were significantly higher in July in three of the teaching units at two hospitals studied compared with the annual mean for that unit. These data suggest that blood glucose control is relatively consistent overall at these hospitals and that measures to improve outcomes with new residents may be warranted.

Our data set has a number of limitations. Point-of-care blood glucose might have been measured multiple times for a given patient when blood sugar was out of target, potentially increasing the total number of events. However, this increased testing would be expected in all cases of severely elevated or low blood glucose, and therefore the data should be consistent across all the months for the hospitals studied. Also, residents may not have had a primary role in managing care of the patients in the study. It is possible that hospitalists, attending nurse practitioners, and physician assistants wrote some insulin orders on the different units. Finally, some interns may not start their inpatient duties until later in their first year, and therefore the possible effect of new residents on percentage of high or low blood glucose events may be distributed in months other than July.

We were not able to prove that residents’ lack of experience is responsible for the higher levels of extreme events observed in three units. Other possible reasons include clustering of very sick patients or patients with difficult-to-control diabetes in July. However, these events are a serious patient care concern, and it would be prudent for program directors and chiefs of service to consider implementation of diabetes management protocols in their hospitals if such a program is not already in place. New physicians should receive training on the optimal use of insulin early in their careers, perhaps during hospital orientation, to help reduce the risk to patients.
